# Qualitative and quantitative research into the development and feasibility of a video-tailored physical activity intervention

**DOI:** 10.1186/1479-5868-8-70

**Published:** 2011-07-01

**Authors:** Corneel Vandelanotte, W Kerry Mummery

**Affiliations:** 1 Centre for Physical Activity Studies, Institute for Health and Social Science Research, Central Queensland University, Building 18, Bruce Highway, Rockhampton, Queensland, Australia; 2 Faculty of Physical Education and Recreation, University of Alberta, W1-34 Van Vliet Centre, Edmonton, Alberta, Canada

## Abstract

**Background:**

Continued low adherence to physical activity recommendations illustrates the need to refine intervention strategies and increase their effectiveness. The purpose of this study was to conduct formative research related to the development of a next generation of computer-tailored interventions that use online tailored video-messages to increase physical activity.

**Methods:**

Five focus groups (n = 30), aimed at males and females, aged between 35 and 60 years, that do not meet the physical activity recommendation, were conducted to allow in-depth discussion of various elements related to the development of an online video-tailored intervention. In addition, a series of questions were delivered to a random sample (n = 1261) of Australians, using CATI survey technology, to gain more information and add a quantitative assessment of feasibility related to the development of the intervention. Focus group data was transcribed, and summarised using Nvivo software. Descriptive and frequency data of the survey was obtained using SPSS 18.0.

**Results:**

Nearly all of the focus group participants supported the concept of a video-tailored intervention and 35.8% of survey participants indicated that they would prefer a video-based over a text-based intervention. Participants with a slow internet-connection displayed a lower preference for video-based advice (31.9%); however less than 20% of the survey sample indicated that downloading videos would be slow. The majority of focus group and survey participants did not support the idea of using mobile phones to receive this kind of intervention and indicated that video-tailored messages should be shorter than 5 minutes. Video-delivery of content is very rich in information, which increases the challenge to appropriately tailor content to participant characteristics; focus-group outcomes indicated a large diversity in participant preferences. 52.4% of survey participants indicated that the videos should be convincing and motivating.

**Conclusions:**

These results provide valuable information to develop an innovative video-tailored physical activity intervention. The results support the feasibility of such intervention, both in terms of users being ready to participate in it, as well as from a point of view whereby current internet infrastructure is able to cope with the demands of downloading videos. Though promising, a number of specific challenges in the development of these interventions were identified (e.g. the videos need to be short, made professionally, and tailor to a larger number of variables) and will need to be overcome in the development and evaluation of this new type of physical activity intervention.

## Background

Despite the vast amount of evidence about numerous health benefits related to participation in regular physical activity [1], over half of the populations in Western countries are not meeting the physical activity guidelines [2-4], which recommend engaging in at least 30 minutes of moderate intensity physical activity a day [5,6]. Although a wide range of intervention strategies to increase physical activity at a population level have been developed and implemented [7-9], the prevalence of inactivity illustrates the continued need to refine interventions strategies and increase their effectiveness. As such, interventions that provide individually 'tailored' information have shown a promising track record of efficacy [10-12].

Tailored physical activity interventions provide participants with individually adapted feedback about their physical activity level and habits [13]. An intervention is 'computer-tailored' when it uses a computerised 'expert system' to generate the personal relevant intervention materials. These interventions can provide large numbers of people with individualised behaviour change information at low cost [14]. Computer-tailored interventions of the *'first' *generation are generally print-based: when the intervention team receives a completed assessment questionnaire a computerised expert system is used to generate printed feedback, which is usually delivered to participants via post. It can take days to weeks to generate and deliver this individualised advice [15]. *'Second' *generation computer-tailored interventions go a step further and deliver immediate personalised feedback via the computer screen. These interventions are usually provided via CD-ROM, intranet (e.g. in a workplace network) or the internet; after completing a computerised survey the text-based personal feedback is displayed immediately on screen allowing participants to save, forward or print the information as they wish [15,16]. Second-generation interventions have advantages over print-delivered interventions: they are highly interactive, immediate and have high reach and availability when delivered via the internet [17]. Individually-tailored interventions of the 'first' and 'second' generation have proven to be significantly more effective than non-tailored (generic or targeted) print- or web-delivered physical activity interventions [12,18-20]. Compared with non-tailored messages, tailored messages are more likely to be read and remembered, saved and discussed with others, and perceived to be interesting, personally relevant and to have been written especially for the respondent [21,22].

However, information and communication technologies have continued to evolve. In recent years the ability and power of internet-based applications have vastly changed and improved. The newest generation of internet-based applications (Web 2.0) is aimed at giving users control of how information is generated, created and shared, by allowing them to upload text, links, videos or photos to popular websites that link in with their personal networks [23]. User statistics show that these new applications are extremely popular and are adopted by vast numbers at an astonishing speed. Examples of popular Web 2.0 applications are social networking sites (e.g. *'Facebook'*), knowledge (e.g. *'Wikipedia'*), photo (e.g.*'Flickr'*) and video (e.g. *'You Tube'*) sharing websites. For example, a 2009 study indicated that nearly 70% U.S. adults have used the internet to watch or download a video, and that watching videos outranks many other online activities (such as using a social network site) [24].

The use of the internet itself has also continued to grow. In 2008-2009 62% of Australian homes had broadband internet access (only 10% of Australian internet users continues to use a dial-up connection), a four-fold increase over the past five years [25]. The increased abilities and uptake of internet technology provides new and enhanced opportunities for behavioural health interventions [26], and have put the foundations in place for the development and evaluation of a next generation of computer-tailored interventions that incorporate the use of these new internet-based technologies [27]. Moreover, in order to avoid that health behaviour change websites attract few (returning) visitors and are being ignored on the ever expanding and evolving internet, behavioural scientists are forced to develop new and innovative interventions that fit with how people use internet today.

Whilst second generation website-delivered computer-tailored interventions haven't been around for very long, they are already becoming outdated as they use the internet in a very basic way. Although individualised and interactive, all they do is provide text on a website [21]. Driven by user demand [28], however, website content is increasingly more often provided in graphical or video format. This development is fuelled by the observation that many people don't like to read big blocks of text on the internet: increasingly often they scan a text for relevant keywords and then quickly move on to something else [29]. Hence, today second-generation interventions have become less appealing to users and, although they have shown to be successful at behaviour change [19,30], problems with attracting, engaging and retaining participants have frequently been reported [7,21,31]. To increase the appeal of future internet delivered computer-tailored interventions they could provide feedback using rich and engaging video-messages rather than simple and plain text-based messages [32]. Although there are no technical limitations to the development of such next-generation interventions, to our knowledge very few have been developed and evaluated within a research context. Video messages have been used in previous health behaviour change interventions; however the videos applied were used to support other (computer-tailored) intervention components, and as such they were generic and not tailored to individual recipients [33-37]. To our knowledge only one study previously applied tailored video-messages [38]; however in this 'interactive multimedia' study on dietary habits combined the use of computer-tailoring, audio, print, graphics and video and it was not possible to determine efficacy according to specific intervention components such as video. The extent to which potential users would be interested in such intervention and how feasible their development is remains unstudied.

According to Danaher et al., one of the more impressive features of internet-based behaviour change programs is their ability to incorporate information rich media components such as video [32]. Therefore, the aim of this study was to quantitatively and qualitatively assess the potential usability and the feasibility of developing a next generation computer-tailored physical activity intervention, which will use individually tailored video-messages to deliver physical activity information to people via the internet.

## Methods

This multi-method study applied focus groups (qualitative) and a state-wide survey (quantitative) to inform the development of a video-tailored physical activity intervention.

### Focus groups

Focus groups were conducted to allow in-depth discussion of various elements related to the development of an online video-tailored intervention (e.g. about the concept of video-tailoring and the optimal length and number of videos). A similar research design has been applied in comparable studies [39].

#### Participants

Participants were recruited from large databases held by the Population Research Laboratory (PRL) at Central Queensland University in March and April 2010. The databases consist of individuals who in previous PRL surveys indicated their willingness to participate in future health related studies. Potential focus group participants were approached and informed about the study via e-mail. To be eligible potential participants had to be aged between 35 and 60 years, not meet the recommendation of 30 minutes of physical activity a day, have no physical or medical barriers to increase their physical activity and speak English. As such it was aimed to recruit people similar to those for whom a video-tailored physical activity intervention would be most relevant in terms of preventing the development of non-communicable chronic disease [40]. Recruitment was also focussed on recruiting an equal number of males and females. To enhance recruitment a $30AUD incentive was provided for those who participated. Written informed consent and demographic characteristics were obtained from participants by means of a short questionnaire prior to each focus group. Participation was completely voluntary, and participants were informed that they could withdraw anytime throughout the session.

#### Procedure

All the focus groups were organised by the PRL and conducted at the university campus. The focus groups were conducted in an empty comfortable and neutral room and snacks and light refreshments were available during the sessions. The sessions were facilitated by two researchers familiar with the topic and aims of the study. One researcher moderated the session, which included explaining purpose and procedure of the focus group sessions, introducing the topic, leading the discussions, encouraging that participants expressed their view even if it differed from others and ensuring that participants were aware that there are no right or wrong opinions. The other researcher co-moderated the sessions, which included handling logistics, taking notes and monitoring recording equipment.

Prior to conducting the focus groups the researchers had developed a focus group discussion guide. This guide was developed in a way that would promote open discussion, and provided prompts to stimulate participants to talk and reengage whenever the discussion stopped (the headings within the *Focus Groups Outcomes *section reflect the topics discussed during the focus groups). All the focus groups were audio-recorded. Following completion of five focus groups (FG1 to FG5) the researchers agreed that saturation had been attained (when applying the method described by Guest et al. [41], only 11% of new quotation codes were assigned to FG5) and that conducting more focus groups would not result in new and meaningful information. The study was approved by the Human Research Ethics Committee of CQUniversity Australia.

#### Analysis

Participant demographic data was analysed using descriptive statistics in SPSS 18.0. Following each of the sessions the audio-recordings were professionally transcribed; they were subsequently checked for quality by a member of the research team. QSR NVivo qualitative analysis software (QSR International Pty Ltd, Melbourne, Australia) was used to organise and manage the data. A qualitative content analysis was conducted, whereby two members of the research team first independently read the transcripts to identify the common themes arising from the focus groups, which were discussed and agreed upon [42]. The data was then organised according to the themes; inconsistencies were once again discussed and revisions were made as needed. Subsequently, summaries of each theme were produced by one researcher, and checked for accuracy by the other researcher.

### Queensland Social Survey

#### Participants and Procedure

A series of questions related to the development of a video-tailored physical activity intervention were included in the annual Queensland Social Survey conducted by the Population Research Laboratory at the Central Queensland University in Australia. The CATI (Computer Assisted Telephone Interview) survey was conducted in July and August 2010 by trained interviewers. A two-staged stratified sampling design was used to randomly select households and individuals in Queensland (Australia) and was designed to reflect the characteristics of the population by the most recent Australian Census. All respondents were 18 years of age or older at the time of the survey, and were living in a dwelling unit that could be contacted by a direct-dialled, land-based telephone service. The sample was drawn from commercially available Electronic White Pages using a computer program to select, with replacement, a simple random sample of phone numbers. Within each contacted household, one eligible person was randomly selected to act as the respondent for the interview. Up to five follow-up calls were scheduled to non-answered numbers. Participants provided informed consent at the start of the CATI survey and approval from the Human Research Ethics Committee of CQUniversity Australia prior to data collection.

#### Measures

A set of new questions, based on focus group outcomes reported in this manuscript and dedicated to the concept of a video-tailored physical activity intervention, was developed specifically for this study and then added to the larger annual Queensland Social Survey (other topics in this survey were: demographics, internet use, nutrition, physical activity, smoking, physical and mental health and social capital). As such this study was able to benefit from the trained CATI interviewers and the well-established and reliable protocols of a state-wide study that has been conducted for nearly a decade. The questionnaire was pilot-tested on a total of 52 randomly selected households in South East Queensland. Interviewer comments (e.g. confusing wording, inadequate response categories, question order effect, etc.) and pre-test frequency distributions were reviewed to assist modifications to the questions. A simplified version of the multiple choice questions and answers categories can be found in Table 1. In order to make it easier for survey respondents to understand what the questions were about a brief introduction (read to participants by a CATI interviewer) was provided to explain why physical activity interventions are needed (regardless of whether or not it is applicable to them or whether or not they would ever take part in such an intervention), but also about how personally relevant or 'tailored' information can be generated using the internet.

**Table 1:** Queensland Social Survey responses (%) to questions regarding computer-tailored physical activity video-messages for total group and stratified for gender and age (N = 1087)

	Total (N = 1087)	Male (N = 539)	Female (N = 584)	-40 (N = 294)	40-60 (N = 537)	+60 (N = 394)
**Through which internet delivery method would you prefer to receive personalised physical activity information?**						
Text (that can be printed)	47.6	47.5	47.6	48.6	49.1	43.6
Voice (MP3 or Pod-cast format)	5.3	4.8	5.8	4.7	5.7	5.4
Video (to watch online or download)	35.8	33.8	37.8	43.5	36.7	26.4
**Why would you prefer a personal video message if that is your preference? (N = 389)**						
It would be more interesting than reading text	18.5	22.5	15.0	26.4	12.9	20.3
It would be quick and easy to view a video online	20.1	17.6	22.2	17.4	23.2	16.2
I would find it easier to understand than reading	34.2	35.2	33.3	31.4	34.5	37.8
It would make me feel more motivated to get active	21.1	18.1	23.7	21.5	20.1	23.0
**How long can a personal physical activity video message be before you lose interest?**						
1 minute or less	13.1	15.2	10.9	11.9	13.6	13.2
1-5 minutes	44.3	42.9	45.9	47.5	46.9	36.4
5-10 minutes	14.3	14.6	13.8	17.6	12.8	13.6
10-20 minutes	9.6	7.3	12.1	10.4	9.5	9.3
More than 20 minutes	6.7	6.6	6.7	7.6	6.8	5.7
**Who would you prefer to present this personal physical activity video to you?**						
Someone like you (same gender, age, etc)	25.6	20.2	30.8	24.1	26.8	24.6
A role model, like a sporting personality	11.5	16.0	7.1	16.3	12.5	3.9
A real activity expert, like an scientist or academic	20.4	19.7	21.2	19.7	20.2	20.4
Someone who looks like a coach or instructor	20.0	18.7	21.2	23.0	19.5	17.9
Doesn't matter	11.5	12.6	10.4	9.7	12.5	11.4
**Would you prefer the personal physical activity video message to be:**						
Factual and serious	12.2	15.8	8.8	12.6	11.5	13.2
Humorous and fun	26.4	25.2	27.6	23.0	29.1	24.6
Convincing and motivating	52.4	48.1	56.8	62.2	52.9	41.8
**How fast/slow can you download videos through your internet connection?**						
Downloading would be 'not slow, fast or very fast'	68.9	68.2	69.5	84.2	69.8	52.2
Downloading would be 'slow or very slow'	19.3	18.9	19.7	13.7	22.3	19.3
**Would you like receiving personal physical activity videos via your mobile phone?**						
I can't view or download videos with my mobile	9.0	9.3	8.7	11.9	8.5	7.2
Yes, great idea	11.0	10.4	11.7	18.7	10.2	5.0
No, I don't like this idea	60.7	59.6	61.9	52.5	65.0	60.7
No, it is too costly to download videos to my mobile	8.7	8.9	8.6	13.7	8.1	5.0

Note: - 'I don't know', 'No response' and categories with very low numbers were not tabulated in order to save space, hence the numbers don't add up to 100%.

- The total number of survey respondents was 1261. Only those with internet access (N = 1087) answered the above questions. Among internet users the second question was only asked to those who preferred video-tailored messages in the first question (N = 389).'

#### Analysis

Data were cleaned and tabulated using SPSS 18.0. The data cleaning process included wild code, discrepant value, and consistency checks. The survey data were analysed using descriptive and frequency statistics. Please note that in the interest of space and to make it easier to interpret Table 1 some of the original answering categories were merged or not reported when they were less relevant (e.g. the 'I don't know' and 'No response' answering categories, as well as categories with very low response numbers were not reported). As such, the reported categories are not always equal to 100%.

## Results

### Participants

Of the 67 people initially contacted 30 participated in a total of five focus groups, which by average lasted 1 hour and 18 minutes (range: 1hr07min - 1hr39min). The numbers of participants for each focus group ranged from 5 to 8. A total of 1261 people participated in the Queensland Social Survey, of which 1087 had access to the internet and were included in this study. The overall response rate for the survey was 35.2% and the estimated sampling error (at 95% CI) was 2.7%. Participant characteristics for both the focus groups and the survey can be found in Table 2. Comparable proportions were reported for gender (46.7% vs. 49.6% was female), educational attainment (53.3% vs. 53.8% had more than high school education), internet access at home (93.3% vs. 88.5%), home internet use (8.7 vs. 7.6 hours per week), and BMI (29.1 vs. 30) for both focus groups and survey participants respectively. However, a larger proportion of focus group participant was overweight or obese (75.9%), employed (86.7%), insufficiently active (83.3%) when compared to the survey participants. Average age was higher in survey participants (52.8 ± 16.3) when compared to focus group participants (47.4 ± 7.1).

**Table 2:** Population characteristics (% or Mean ± SD), for focus group and survey participants

	Focus Groups (N = 30)	Queensland Social Survey (N = 1261)
Females	46.7	49.6
Age (years)	47.4 ± 7.1	52.8 ± 16.3
More than high school education (university/ technical)	53.3	53.8
Part or full time employed	86.7	59.0
Income Higher than $52,000 a year	53.8	26.8
Home internet use (hrs/wk)	8.7 ± 11.0	7.6 ± 11.5
High confidence using internet	64.0	-
Internet access at home	93.3	88.5
Overweight/obese (BMI ≤ 25)	75.9	65.6
BMI	29.1 ± 6.6	30.0 ± 14.6
Wants to become more active	90.0	-
Insufficiently Active	83.3	53.3

### Focus Group outcomes

#### What do you think of the concept of video-tailoring?

After explaining the concept of 'video-tailoring' a large majority of focus group participants (26 out of 30) agreed that the concept was good and interesting; they also indicated that they would be happy to receive a video-tailored intervention themselves (*'Its fantastic!' *- F, age 51, FG2). They supported the concept of a program that adapts to individual situations, that is personally relevant and which eliminates redundant information. Participants liked the anonymity of such program rather than having to interact with a 'real' person, and indicated that under such circumstances they would likely to be more honest about real physical activity levels. Participants saw the use of tailored-videos as a natural evolution of how the internet is currently evolving and pointed out that people prefer watching a video over reading text.

#### How long can the video-tailored messages be?

The vast majority of participants (28 out of 30) agreed that the video-messages should be short and ranging between one and maximum five minutes (*'short, sharp and to the point' *- M, age 39, F4). Many people (12 out of 30) seem to prefer a system whereby the very first tailored video-message would be designed to attract attention and engage people into the program; and that therefore it should be very short, 'upbeat' and 'snappy'. After this initially short video longer more detailed messages could be presented when participants would already be engaged with the program. How long videos can be also depends on how interested people would be in their own physical activity levels and how personally relevant the information is: the higher interest levels and relevance the longer a video can be. Videos can also be longer if participants know in advance how long it will take to watch it.

#### How many video-tailored messages can there be?

Participants had difficulties outlining the number of video-tailored messages that would be acceptable ('*three perhaps, mounting up to a total of 15min of video*' - F, age 55, FG1). Some participants (5 out of 30) pointed out it would be good if they could 'tailor' how many videos one gets, based on feedback they provide about this. A large majority of participants (25 out of 30) think it would be a good idea not to provide all videos at once, but rather one per week. In this way one could cope with more videos and information. According to the participants providing all videos at once might also cause people not to return to the website if they had a look at everything already. Participants also indicated that there should be a follow-up video a while after the program has finished *('a booster to get people back on track' *- F, age 50, FG3).

#### Who should be in the video tailored messages?

Participants were ambivalent about who should be in the videos to present the physical activity information to the viewer, often with opposing opinions. However, the main underlying idea supported by most participants (22 out of 30) was that whoever presents the video needs to be credible and convincing ('*someone that is very fit would not be credible for everybody' - *M, age 35, FG2). Therefore people that can talk from experience about the topic would be much liked. Many suggested (11 out of 30) that the option of self-selecting the video presenter would be a feasible approach ('*pick your presenter' *- M, age 44, FG1). Many also liked the idea of having several different presenters in the video according to the topic ('*a GP for medical topics or a coach for getting more active' - *M, age 53, FG5). Some participants would like to see role models, celebrities, famous people, sports stars; others don't and would be just happy with anybody as long as they would be 'real', passionate and motivating. Some participants would like to see similar ages, sexes, ethnicities and weight, whilst others don't care about it or would even prefer the opposite. Most (27 out of 30) agree that an overweight presenter wouldn't be very convincing. A surprisingly high number of participants (9 out of 30) liked the idea of using cartoons or animations, reporting them as 'neutral' presenters. The participants didn't like the idea of professional actors ('*they get paid to fake it*' - M, age 54, FG3). A researcher or professor would be credible, but needs to avoid being condescending.

#### To what level of detail and to which variables should the video messages be tailored?

Initially participants struggled to give specific answers to this question, but agreed that some tailoring is better than no tailoring at all. ('*The more tailoring, the more interesting! One size fits all is no good!' *- M, age 40, FG4). Answering about 20 questions would be the limit in order to receive personally relevant feedback. Several participants suggested that one should be offered the option to choose: the approach (e.g. factual vs. humours), who presents, number of questions needed to answer in order to receive feedback, and at what interval new messages should be delivered. Messages should also be tailored according to personal background, age, attitudes, barriers to be active, and motivation to become more active.

#### Which mode of delivery do you prefer for the video messages: website or mobile?

The majority of focus group participants (24 out of 30) would prefer receiving the tailored video-messages via the internet and not via their mobile phone ('*that is for young people' - *M, age 39, FG1). However, participants were supportive of receiving very short video-messages on their mobile phone (e.g. 20 seconds) as a reminder or to give a tip on being active. In line with this, most participants (21 out of 30) would also not mind receiving reminders (SMS or e-mail) via their phone when a new tailored video-message would be available on the website. Reasons mentioned for not being supportive of phone delivery were: associated download cost of receiving videos via a mobile phone, receiving messages when not being in the 'right' environment making it hard to pay the attention that these messages disserve, the phone screen is too small and the quality of the videos would have to be low to allow downloading them.

#### How professional should the video messages be?

Most participants (19 out of 30) agreed that the level of professionalism doesn't have to be the highest achievable level; however they also agreed that the video messages will need to look professional in order to be credible and engaging. It would be more important to work with a professional production team behind the camera (light, sound, camera), than who is in front of the camera. Use of a studio would be recommended, but not exclusively supported (*'also shoot at everyday locations used by 'real' people' *- F, age 60, FG3).

#### What approach should the video messages have?

Participants found it difficult indicating what approach the video-messages should have (*'ideally it is individually tailored, if that would be possible' *- M, age 43, FG5). However, they indicated that the messages should be positive, friendly, encouraging, motivating, passionate, caring, engaging and inspiring; but at the same time not be too 'preachy', 'pushy', confronting or 'hard' (*'it will create resistance to the message' *- F, age 51, FG2). A light hearted or humorous approach would be appreciated by many participants, but participants acknowledge that this would be difficult to achieve as everybody has a different idea of what is 'funny'. The 'tempo' of the videos should be 'upbeat' ('*keep it moving' *- M, age 49, FG1), it shouldn't be neither too fast nor too slow and depending on the topic tempo variations are encouraged (*'to prevent people from phasing out' *- F, age 47, FG4). A 'negative medical approach' (*'scare techniques' - *F, age 43, FG1) would not be recommended ('*although some topics require you to be more serious than others' *- M, age 39, FG4).

#### What kind of graphs and animations should be used in the video-tailored messages?

The majority of participants (24 out of 30) were supportive of using graphs, animations and occasionally text into the video messages; they claim it would be more interesting, motivating and engaging to watch videos that incorporate features like that (*'it would take longer to get bored and switch off' *- F, age 53, FG2). However, the graphs would need to be simple, basic, and easy to understand; and there shouldn't be too many of them and they should be well explained.

#### How would you like the video messages to be stored?

Most focus group participants (22 out of 30) would like their personal video messages to be saved onto a personal section of the website so that they would be able to view them again later (*'the website needs to be secure though' *- F, age 44, FG3). In addition, most people (19 out of 30) would also like to have the ability to store the newly generated video on their personal computer hard drive, so that they would not have to download it again if they want to watch it again. This would also make it easier to share it with friends or family via e-mail or social networking sites. Most participants (24 out of 30) preferred the option whereby they would be able to receive the personalised videos an unlimited amount of times, to see whether they would have progressed over time, or just to see what would happen if you answer some questions differently.

#### Will your internet connection cause problems for downloading the video-tailored messages?

Most of the focus group participants (20 out of 30) indicated to have a broadband connection, but nonetheless indicated that slow download times should be avoided if possible (*'nobody likes to wait and you might lose people if it takes too long' *- M, age 43, FG5). Watching videos would not be an option with a dial-up connection, and in this respect low size videos were encouraged by participants. Participants strongly indicated that they would prefer to wait a little longer whilst the video downloads over having to watch a video that is stopping and starting all the time; in this respect they also mentioned that an accurate progress bar ('*that shows how much longer it will take for the download to complete*' - F, age 56, FG2) would be needed, and 'something' to keep one distracted whilst waiting would also be a must. The level of patience to wait for a video to download appeared to vary from '*no patience at all'*, to *'I don't mind waiting for 2 minutes while it loads'*.

### Queensland Social Survey outcomes

Participant survey responses to questions regarding computer-tailored physical activity video messages can be found in Table 1. Overall participants would mostly prefer to receive computer-tailored physical activity messages in text (print) format (47.6%), although a large proportion of participants would like to receive video-messages (35.8%). Receiving voice messages was not popular (5.3%). There were no outspoken gender differences; whereas according to age it was apparent that participants under 40 years of age (43.5%) more often preferred video messages when compared to participants that were over 60 years of age (26.4%). The most important reason for participants to prefer a video-tailored message was that it would be 'easier to understand over reading' (34.2%); and this was especially the case in the oldest age group (37.8%). For the total group, equally important was that it would be more 'interesting' (18.5%), 'quick' (20.1%) and 'motivating' (21.1%) when compared to a personal text-based message. For females (15%) and participants between 40 and 60 years of age (12.9%) the videos being 'more interesting over reading text' was less important when compared to males and older age groups.

When asked who should present the personal physical activity advice most people preferred someone similar to themselves (25.6%), but this was closely followed by a physical activity expert (20.4%) or a 'coach' (20%). For females (30.8%) 'someone like you' was a lot more important than for males (20.2%); however males (16%) preferred a 'role model' a lot more than females (7.1%). Especially 'role models' were less preferred as participant age increased (only 3.9% for those aged over 60 years). Across groups about 10% to 12% indicated not to care about who is presenting the video messages.

A large majority of participants would prefer the videos to be 'convincing' (52.4%), over 'humorous' (26.4%) and 'factual' (12.2%). Especially participants under the age of 40 (62.2%) would like the videos to be 'convincing', when compared to the two older age groups (52.9% and 41.8% respectively). Especially men (15.8%) would like the videos to be factual when compared to women (8.8%).

For the majority of people the video-messages should be no longer than five minutes (57.4%); and of those 13.1% indicated that they should be shorter than one minute. Only 30.6% of participants indicated that the video-messages can be longer than 5 minutes. An overwhelming majority of participants indicated that they can download videos from the internet quickly (68.9%); this was especially the case when comparing younger participants (84.2%) with older participants (69.8% and 52.2% respectively).

Only about 10% of the sample indicated that they would also like to receive personal physical activity videos via their mobile phone (although this is nearly double in the younger respondents (18.7%); whereas the majority of participants (60.7%) didn't like this idea. About 10% of the sample indicated that their mobile phone would not be suitable for viewing videos. Associated cost with downloading videos was mainly a concern in younger respondents (13.7%) when compared to older groups (8.1% and 5% respectively).

## Discussion

The results clearly indicate that a video-tailored physical activity intervention is acceptable for potential users, and that developing such intervention is feasible. Furthermore, a lot of useful information was obtained to inform the future development of this type of interventions. It has often been suggested that internet-delivered interventions that are highly interactive and appealing in use will result in higher participant engagement and retention, and might also result in higher and longer term effectiveness [7,31,32]. Whilst to date there is no direct evidence to support that computer-tailored interventions are capable of increasing engagement and retention [43], they have previously shown to be successful in changing behaviour [11,12] and incorporating this 'next generation' video enhancement (which increases their appeal), is anticipated to further increase their effectiveness. The detailed information presented in this study is entirely focussed on providing assistance with the development of these innovative interventions.

Nearly all the focus group participants supported the concept of a video-tailored intervention, whereas a much lower proportion of survey participants indicated using video would be their preferred method of delivery. This is likely due to the extensive introduction and illustration on this topic received by focus group participants, resulting in higher understanding and appreciation of the concept of video-tailoring when compared to survey participants who only received a brief explanation over the phone. Social desirability might also have contributed to focus group participants showing greater support for video-tailored interventions. However, when compared to an online survey, conducted by Marshall et al., the current support for video delivery of physical activity advice has risen to a level three times higher than what it was in 2003 [44]. The survey, by Marshall et al., identified the preferred sources of advice on how to become more physically active and only 12% of internet users preferred receiving advice using video. As such, only 7 years later, the continued 'internet revolution' has rapidly changed user preferences, with now more than 35% of participants indicating that they would prefer video-based advice. The preference of text over video delivery by survey participants is likely a case of people being unfamiliar with a new and unknown type of intervention (video), and preferring what they know best (text). As internet speed, number of broadband connections and new websites rapidly keep evolving in a direction that is more suitable for video-delivery of content, the number of people that prefer video over text delivery will undoubtedly continue to grow. This is also reflected by having a higher proportion of participants less than 40 years of age who preferred to receive video-tailored advice (43.5%), as younger people are often setting new internet trends which are later adopted by the broader population.

A recent study indicated that those most likely to watch videos on the internet are young, male and have a broadband connection [24]. This is in line with the current study with regards to those who are younger, however in this study more females (37.8%) indicated to prefer video-tailored messages when compared to men (33.8%). This might be due to the health related nature of the video messages, as males typically show less interest in their health when compared to females [45,46]. In this study it was also found that participants with a slow internet connection more often preferred to receive text-based advice (54.7%) compared to participants with a fast internet connection (49.6%). Similarly, those with a fast internet connection more often preferred to receive a video-based advice (40.3%) compared to those with a slow internet connection (31.9%) (Chi^2 ^= 632; P < 0.001; not reported in results section). In relation to this, a study conducted in 2005 showed that bandwidth constrains of 'video-rich' health behaviour change websites were too large to allow satisfactory use with dial-up modems [32]. It is therefore encouraging that broadband connections are overtaking the marked at an astonishing rate, with currently only 10% of Australians continuing to use a dial-up connection [25]. The survey results of this study were in line with this, indicating that for less than 20% of the sample downloading videos would be 'slow' or 'very slow'.

From these results it thus seems that many internet users are ready to receive a video-tailored physical activity intervention, and that current internet infrastructure is able to support it. However, the results from this study also indicate that it is too soon to go yet another step further and use mobile phone technology to implement this type of intervention. Even though 'smart phone' use is on the rise (according to market research, conducted in June 2010, 36% of Australians owns a smart phone and this is likely to increase to 50% within one year), the majority of both focus group and survey participants did not like the idea of using their mobile phones to receive this kind of intervention. Only 10% of survey respondents were in favour of this idea; although this number was nearly double for those under 40 years of age. The reason for the low support might result from the fact that the average age of participants in both the focus groups and the survey was relatively high (47.4 and 52.8 respectively). An American study, conducted in 2010, showed that the number of people that have watched a video on their mobile phone sharply drops with increasing age: 40% have done so in those aged 20 to 30 years, 20% in those aged 30 to 50 years, and only 6% in those aged 50 to 65 years [47]. Thus, although it is too early to implement a video-tailored intervention though mobile phones to date, it is likely that this will change in the future.

The results from both focus groups and survey highlight a number of challenges that health promotion professionals will face when designing video-tailored interventions. Content presented by means of video is very information rich, and as such there are a lot more 'variables' that one can tailor to in order the make the advice more interesting or appealing, when compared to information that is presented in plain text format. On many occasions during the focus groups participants expressed very diverse preferences with regards to, for example, who presents the information in the videos, what approach the videos should have, at what interval new video-messages should be delivered and more. This is in line with the results from the survey which did not identify a clear preference as to whom should present the video-messages. Several focus group participants suggested that one should be offered the choice ('pick your presenter'). Whilst technically feasible, accommodating such preferences would be a huge logistical challenge, as each time a participant is offered such a choice it would double, triple or quadruple (depending on how many choices are offered) the entire database of video-messages that support the program. Unlike writing text, producing videos is difficult (focus group participants indicated that a professional production is required to be engaging and credible), time consuming and expensive. Due to a lack of evidence it is unclear whether accommodating such 'personalisation' of preferences would result in higher intervention effectiveness [48]. However, two studies did examine this and their outcomes suggest that efforts should be made to personalise and tailor feedback on as much variables as feasibly possible. A HIV prevention study provided participants with the option to choose one of four virtual characters to guide them through the intervention, consistent with the focus group outcomes of the present study, preferences for the virtual presenters were very diverse (they were all selected by large proportions of participants) and participants responded very positive towards the intervention [37]. Further, a study by Dijkstra et al. evaluated the effects of 'feedback, 'personalisation' and 'adaptation' in an attempt to uncover the working mechanisms of computer-tailoring, and concluded that both 'feedback' and 'personalisation' (but not 'adaptation') were effective to increase intervention effectiveness [49].

Another challenge is that video-messages need to be short above anything else. Over half of survey participants want the messages to be shorter than 5 minutes; 15% of men want them to be even shorter than one minute. The focus groups yielded similar outcomes with a majority of participants indicating that 5 minutes is the maximum length and that they would not watch messages that are longer. This requirement makes it hard for health professionals. Five minutes might be sufficient to communicate a message, but it is doubtful that this will be enough to change behaviour. Although not much is known about the actual exposure participants have to intervention materials, it is obvious from previous website-delivered physical activity interventions that higher exposure (often measured through number of logins into intervention website) leads to higher intervention effectiveness [7,50]. A potential way of dealing with this challenge might be to develop multiple video messages that are reasonably short. As such, participants would be able to view the different videos available on a website at their own pace, or alternatively they could be provided with new videos at intervals set by health professionals. The focus groups revealed that participants are open to the idea receiving multiple messages at a set interval, which would allow them to receive more advice without causing an information overload.

A major strength of this study is that it combines qualitative focus group data with quantitative survey data, and as such overcomes some of the limitations that are prone to each of these research methods when used separately. For example, focus group participants were a convenience sample and selection bias might have occurred as those approached participated on a voluntary basis. Furthermore, social desirability bias may have emerged by discussing issues in a group, even though participants were encouraged to express their opinion even if it was different from others. However, representativeness issues were overcome by adding and comparing data on the same topic from a large state-wide survey which aimed to reflect the characteristics of the population taken from the most recent Australian Census. In turn, detailed and in depth information about a specific topic cannot be gathered using a large scale survey methods, this is where the focus groups show their added value, with their ability to discover hidden opinions and attitudes through discussions with peers, and their ability to gather extensive information in a relatively short period of time. Finally, one specific limitation with regards to the survey outcomes should be mentioned: a large proportion (up to 20%) of older survey respondents (over 60 years of age) did not answer the questions in relation to personalised video-tailored physical activity messages (not reported in Table 1), whereas this was minimal (generally less than 2%) in younger participants (less than 40 years of age). This is likely an indication that the oldest group of survey participants has not yet fully caught up with new aspects of internet technology, or have more difficulties understanding the concepts in the questions asked. As such, survey outcomes of the oldest group of respondents should be interpreted with caution.

## Conclusions

The results from this research provide valuable information to guide the development of a new and innovative video-tailored physical activity intervention. The results also support the feasibility of such intervention, both in terms of users being ready to participate in it, as well as from a technical point of view whereby the infrastructure available to the majority of internet users is able to cope with the demands of downloading videos. Though promising, a number of specific challenges in the development of these interventions were identified (e.g. the videos need to be short, made professionally, and tailor to a larger number of variables). Hence, the next step will be to examine whether or not these challenges can be overcome, through the development and subsequent evaluation of this new type of physical activity intervention. Finally, the results indicated that it is too soon to implement such intervention though mobile phones.

## Competing interests

The authors declare that they have no competing interests.

## Authors' contributions

CV was involved in conceptualization, study coordination, data collection, analyses and manuscript development; WKM was involved in conceptualization, data collection and analyses. All authors read and approved the final manuscript.
